# How common? Exploring the prevalence and risk factors for *Pseudomonas aeruginosa* in diabetic foot infections among kidney transplant recipients

**DOI:** 10.1017/ash.2025.10232

**Published:** 2025-11-28

**Authors:** Mario Escoriza Gonzalez, Daniel A. Burack, Margaret McCort, Phyu Thwe, Luz Liriano-Ward, Rohan Goyal, Rachel Bartash

**Affiliations:** 1 Department of Internal Medicine, Montefiore Medical Center, Bronx, NY, USA; 2 Division of Infectious Diseases, Department of Medicine, Montefiore Medical Center, Bronx, NY, USA; 3 Department of Pathology, Montefiore Medical Center, Bronx, NY, USA; 4 Division of Nephrology, Department of Internal Medicine, Montefiore Medical Center, Bronx, NY, USA; 5 Division of Infectious Diseases, Department of Medicine, New York-Presbyterian/Weill Cornell Medicine, New York, NY, USA

## Abstract

**Objective:**

Concern for *Pseudomonas aeruginosa* in diabetic foot infections (DFIs) is common and a driving factor for empiric antibiotic prescribing, particularly among immunocompromised hosts. This study sought to identify the prevalence of *Pseudomonas aeruginosa* in DFIs among kidney transplant recipients and the use of antipseudomonal agents for empiric antibiotic coverage.

**Design:**

Retrospective cohort study.

**Setting:**

Large, urban, tertiary care transplant center.

**Patients:**

39 kidney transplant recipients who developed DFIs at our institution between 2015–2023.

**Methods:**

We performed a retrospective review of 47 DFIs in 39 kidney transplant recipients hospitalized for a DFI with a positive tissue culture.

**Results:**

A total of 79 organisms were isolated. *Pseudomonas* represented 6.33% of organisms (5/79) and was present in 5/47 DFIs (10.6%). *Staphylococcus aureus* was the most commonly isolated organism (30.4%, 24/79). Compared to those with non-Pseudomonas infections, patients with cultures positive for *Pseudomonas* were temporally closer to transplant (50.6 days +/- 214.9 vs 1047.6 days +/- 766.4, ***p* = 0.041**) and more likely to have been treated for rejection with the past 12 months (2/5, 40% vs 0/42, 0%, ***p* = 0.009**). Eighty three percent of patients received empiric antibiotic coverage with an antipseudomonal agent.

**Conclusions:**

Our study found a low overall prevalence of *Pseudomonas* in DFIs among kidney transplant recipients. Despite this, empiric coverage with antipseudomonal antibiotics was common, highlighting a potential antimicrobial stewardship target in solid organ transplantation.

## Background

Though concern for *Pseudomonas aeruginosa* (PSA) as a pathogen in diabetic foot infections (DFI) is common, this conception stems from higher rates of PSA in warmer climates and patients with water exposure.^1,2^ Studies in more temperate climates suggest a low prevalence of this organism, ranging from 4.5 to 9%.^3,4^ Furthermore, even when present, the clinical virulence of PSA in DFI has been debated. In clinical studies, patients with PSA isolated from DFIs had similar rates of clinical response to antimicrobial therapies, regardless of whether an antipseudomonal agent was administered, suggesting this organism may represent a frequent colonizer that does not necessarily require targeted therapy.^5,6^

As a result of the low prevalence and questionable virulence, the Infectious Diseases Society of America (IDSA) guidelines on DFI management only recommends empiric therapy directed at PSA for patients with risk factors for pseudomonal infection, including a high local prevalence of PSA, warm climate, and frequent water exposure.^1^ However, despite guideline recommendations, studies have shown that as high as 88% of patients with DFIs receive empiric antipseudomonal coverage.^4^

Management of DFIs is particularly important for kidney transplant recipients (KTR). KTRs typically have prior histories of chronic kidney disease, a population in whom DFI outcomes are worse, with increased rates of amputation and mortality.^7^ KTRs also receive high doses of immunosuppression following transplant, which places them at a higher risk for poor wound healing.^8^ Furthermore, due to concerns for the prevalence of multidrug-resistant infections in immunocompromised hosts and their potential outcomes on allograft function,^9,10^ KTRs often receive more frequent and broader courses of antimicrobials, potentially leading to colonization with more antibiotic-resistant organisms.^11^

Despite the above risk factors, some studies have demonstrated comparable outcomes of DFI in KTRs as compared to non-KTRs, including similar rates of amputation and mortality.^12^ These conflicting data merit further investigation into DFIs in KTRs, with a particular focus on the prevalence of PSA to guide appropriate antimicrobial therapy, as to our knowledge, no studies have specifically evaluated the prevalence of this organism in KTR DFIs. Additionally, judicious use of antibiotics in this patient population is crucial to prevent the development of multidrug-resistant infections.

A review of all patients with DFIs performed at our institution found the local prevalence of *P. aeruginosa* to be low at approximately 10%.^13^ We hypothesized the prevalence of PSA in DFIs in our KTR population to be similarly low and aimed to understand risk factors for this organism as well as empiric prescribing patterns for DFIs in our KTR population.

## Methods

We conducted a retrospective chart review of patients >18 years old with a history of kidney transplantation who were treated for a DFI between January 2015-December 2023 at our tertiary care medical center. KTRs with DFIs were identified by cross-referencing a list of patients who had undergone kidney transplantation between 2015–2023 and were actively followed at our institution with a list of cultures gathered by the Microbiology department. Patients were included if the positive cultures were obtained from a DFI during the study period (2015–2023). Cultures included wound, tissue, and/or fluid collections obtained either surgically or at the bedside. Patients were excluded if they were: treated empirically for DFI without obtaining cultures, had negative cultures, treated as outpatients, had concomitant infections unrelated to their DFI affecting antibiotic selection, and/or were not actively followed by our transplant clinic. Patients could be included for more than one infection if their repeat infection occurred at a non-contiguous site or >6 months after their initial infection to prevent including recurrent or refractory DFI which may be associated with higher rate of resistant organisms, including PSA.

After excluding patients who did not meet eligibility, we determined the prevalence of each pathogen identified, including PSA. We then performed a retrospective cohort study comparing two groups: (1) those with cultures positive for PSA, and (2) a “control” group, including any patients with cultures positive for a non-PSA organism. The characteristics of patients in both cohorts were compared to identify potential risk factors for PSA. Data collected included: demographics, medical comorbidities (and Charlson Comorbidity Index), immunosuppressive regimen, receipt of rituximab or thymoglobulin in prior 12 months, treatment for graft rejection in prior 12 months, previous isolation of multidrug-resistant organism (MDRO) from any culture, history of prior PSA infection from any site at any time in the past as recorded per EMR, antibiotic use for any reason in the month preceding DFI, and hospitalization in the 6 months prior to DFI.

Additionally, the following data were collected regarding the patients’ DFI and hospital admission: microorganism(s) isolated on wound culture, presence of severe DFI defined per IDSA guidelines (local infection with >2 of the following: temperature >38C or <36C, HR>90 beats/min, RR > 20 breaths/min, PaCO2 < 32 mmHg, WBC count> 12000 or <4000 cells/uL or >10% bands),^1^ presence of bacteremia secondary to DFI, and empiric antibiotics used for DFIs prior to microorganism identification. The following antibiotics were considered to have antipseudomonal activity: aztreonam, cefepime, ceftazidime/avibactam, ceftolozane/tazobactam, cefiderocol, ciprofloxacin, levofloxacin, meropenem, meropenem-vaborbactam and piperacillin/tazobactam.

The primary outcome of the study was the prevalence of DFI secondary to PSA in KTRs. Secondary outcomes included the frequency of empiric antipseudomonal antibiotics, as well as identifying clinical predictors of PSA infection.

Descriptive statistics were reported using standard summary statistics: frequencies/percents, means/standard deviations, or medians/interquartile ranges, where appropriate. Bivariate associations between variables were assessed using χ2 tests, Fisher’s exact tests, Student’s t tests, logistic regression, or analysis of variance tests, where appropriate. Multivariate regression analysis was performed to adjust for potential confounder variables. All tests were 2 sided and p-values < 0.05 were considered significant.

## Results

Forty-seven DFIs occurring in 39 KTRs were included. The median age was 62 and the majority (84.6%) of KTRs were men. The average time from transplant to DFI was 1,098.3 days. Fifteen DFIs (31.9%) had a prior history of PSA infection, 16 (34.0%) had a prior history of infection with a multidrug-resistant organism, and 20 (42.6%) received antimicrobials in the 30 days preceding DFI. Additional demographic data is shown in Table 1.

**Table 1.** Demographics, risk factors, and outcomes of kidney transplant recipients with diabetic foot infections secondary to *Pseudomonas aeruginosa* compared to other organisms

	N = 47 DFI total, 39 unique patients*	Pseudomonas (*n* = 5)	Non-Pseudomonas (*n* = 42)	Odds ratio [95% confidence interval]	*P* value (or Fischer’s exact where appropriate)
**BASELINE DEMOGRAPHICS AND VARIABLES**					
Age Average Median +/- SD	59.9 62 +/- 9.7	—	—	–	–
Age category 34–49 50–59 60–69 70–79	6 15 19 7	1 0 2 2	5 15 17 5	1.110 [0.607, 5.967]	0.223
Sex* Male Female	33 6	5 0	28 6	0 [0, 4.18]	0.307
Race^*†*^ 1= Black or African American 2 = White 3 = Other 4 = Asian 5 = Decline to answer 6 = Other Pacific Islander	12 7 17 1 1 1	2 2 1 0 0 0	10 5 16 1 1 1	0.600 [0.224, 1.607]	0.507
Ethnicity^*†*^ 1 = Hispanic/Latino 0 = Not Hispanic/Latino 8 = Declined to answer	21 17 1	1 4 0	20 13 1	0.207 [0.021, 2.007]	0.261
Type of organ transplant^*†*^ Kidney Kidney-liver Kidney-pancreas	33 2 4	4 0 1	29 2 3	0.952 [0.165, 5.492]	0.588
History of HIV^*†*^	1	0	1	–	1.000
History of malignancy^*†*^	8	8	0	0 [0, 3.699]	0.563
History of PAD^*†*^	34	5	29	–	1.000
History of connective tissue disease^*†*^	5	1	4	2.375 [0.387, 33.597]	0.517
History of MI^*†*^	10	2	8	2.833 [0.199, 28.616]	0.587
History of CHF^*†*^	16	2	14	1.333 [0.099, 13.016]	1.000
History of COPD^*†*^	5	0	5	–	1.000
History of dementia^*†*^	5	0	5	–	1.000
History of peptic ulcer disease^*†*^	2	0	2	–	1.000
Time from SOT to DFI culture (mean +/- SD)	1 098.3 days +/- 724.7	50.6 days +/- 214.9	1 047.6 days +/- 766.4	–	0.041*
Polymicrobial cultures Single organism in culture	21 26	4 1	17 25	5.88 [0.504, 300.97]	0.158
Charlson comorbidity index >/=8	27	4	23	3.304 [0.287, 171.122]	0.377
Antibiotics within prior 30 days	20	3	17	2.21 [0.33, 14.64]	0.638
Treatment for rejection in prior 1 year	2	2	0	–	0.009*
Thymoglobulin in prior 1 year	5	2	3	8.667 [0.492, 108.476]	0.081
History of MDRO	16	2	14	1.333 [0.099, 13.017]	1.000
History of Pseudomonas infection	15	3	12	3.625 [0.355, 47.085]	0.311
Hospitalization in prior 6mos	32	4	28	2 [0.173, 105.412]	1.000
Anatomic source of infection Left foot Left toe Right foot Right toe Right metatarsal bone Right heel bone	13 11 8 12 2 1	3 0 0 2 0 0	10 11 8 10 2 1	0.758 [0.358, 1.604]	0.422
MMF at time of DFI	38	5	33	–	0.567
Tacrolimus levels (*n* = 44) Mean Std error Std dev. 95% CI		6.76 1.317 2.944 [3.10, 10.417]	7.02 0.722 4.509 [5.56, 8.48]	–	0.900
GFR at time of DFI Mean +/- SD	52.19 (+/- 24.90)	55 (+/- 18.19)	51.86 (+/- 25.73)	–	0.793
A1c at time of DFI (mean +/- SD), n = 46	8.34 (+/- 1.95)	8.66 (+/-2.89)	8.3 (+/- 1.85)	–	0.702
**OUTCOMES**					
Osteomyelitis present	34	3	31	0.532 [0.054, 7.268]	0.607
Severe DFI	23	1	22	0.227 [0.004, 2.626]	0.348
Fever	14	1	13	0.558 [0.011, 6.476]	0.528
Death within 1 year of DFI	1	0	1	–	1.000
Bacteremia	10	0	10	–	0.569
Graft failure within 1 year of DFI	1	0	1	–	1.000

^†^Unique patients were only included once (excluding duplicate DFIs for same patient) (*n* = 39 unique patients).

DFI, diabetic foot infection; SOT, solid organ transplantation; MDRO, multidrug-resistant organism; MMF, mycophenolate; PAD, peripheral artery disease; COPD, chronic obstructive pulmonary disease; CHF, congestive heart failure; HIV, human immunodeficiency virus.

**Figure 1.**
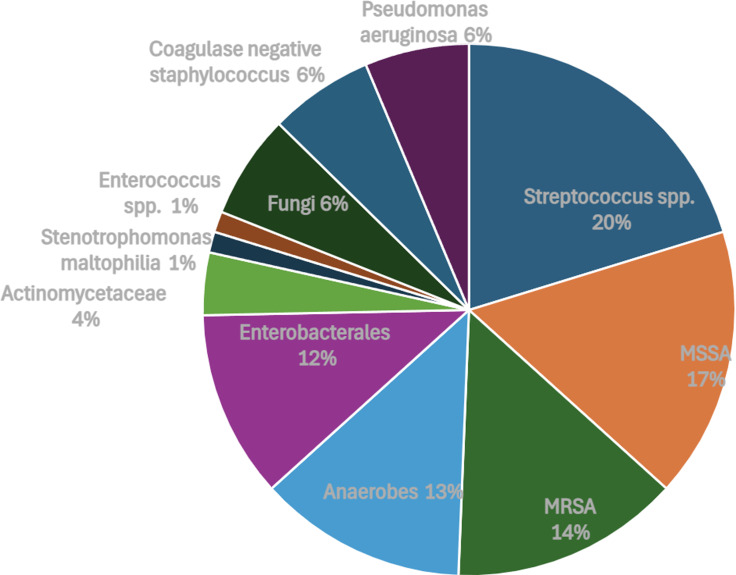
Organisms isolated from diabetic foot infections (*n* = 79).

Of the 47 DFIs, 79 microorganisms were isolated, with 44.7% (21/47) of infections being polymicrobial. PSA represented 6.33% of organisms isolated (5/79) and was present in 5 of 47 DFIs (10.6%). *Staphylococcus aureus* was the most commonly isolated organism, representing 30.4% (24/79) of all isolated organisms, and was present in 24 of 47 DFIs (51.1%). Among *Staphylococcus aureus* isolates, 45.8% (11/24) were methicillin-resistant. *Streptococcus* species were the next most common and represented 20.3% (16/79) of organisms. Thereafter, anaerobic organisms were the most commonly isolated, including *Bacteroides* (5/79, 6.33%), *Prevotella* (4/79, 5.06%), *Actinomyces* (3/79, 3.80%), and *Cutibacterium acnes* (1/79, 1.27%). Additional microbiologic data is shown in Figure 1.

The majority (72.3%, 34/47) of DFIs had underlying osteomyelitis at time of diagnosis, 23 DFIs (48.9%) met criteria for severe DFI and 10 DFIs (21.3%) had concomitant bacteremia. Eighty-three percent of DFIs (39/47) received empiric antimicrobial therapy with antipseudomonal coverage. Graft failure and/or death within 1 year of DFI occurred in one patient (DFI cultures were + for methicillin-resistant Staphylococcus aureus).

Patients with DFI culture positive for PSA were compared to those with cultures positive for other organisms via statistical analysis. DFIs secondary to PSA occurred earlier after SOT compared to DFIs caused by other organisms (50.6 d +/- 214.9 vs 1 047.6 d +/- 766.4, ***P* = .041**). Treatment for rejection in the year prior to DFI was more common in the PSA cohort (2/5, 40%, vs 0%, ***P* = .009**). A similar trend was seen for thymoglobulin receipt in the year prior to DFI (2/5, 40%, vs 3/42, 7.14%, *P* = .081), but this was not statistically significant.

No other factors, including age, sex, Charlson comorbidity index, or other comorbidities, were associated with a significant difference in rates of PSA (Table 1). Receipt of antibiotics within 30 days prior to DFI, hospitalization within 6 months prior to DFI, and history of prior PSA infection were not significantly associated with PSA-positive DFI. Clinical outcomes, including osteomyelitis (*P* = .607), severe DFI (*P* = .348), bacteremia (*P* = .569), and graft failure or death within one year of DFI (*P* = 1.000), were also not significantly different between the two cohorts. Additional results are shown in Table 1.

## Discussion

Our retrospective review of DFIs in KTRs found the prevalence of PSA to be low at 6.33%. This prevalence is consistent with comparable data reported in the non-transplant population, including that from our own institution.^3–4,13^ These findings are particularly useful in guiding antibiotic management decisions when culture data is not available, a not uncommon scenario, particularly if there is no draining wound or planned surgical debridement.

Despite the low prevalence, empiric use of antipseudomonal antimicrobials in our population was high at 83%. This study highlights a potential opportunity for antimicrobial stewardship in our kidney transplant population, aimed at limiting universal empiric use of antipseudomonal agents given its low prevalence. Our institution recently published results highlighting improved empiric prescribing for DFIs after implementing an electronic medical record (EMR) order set.^13^ Following the EMR intervention, empiric use of antipseudomonal agents decreased from 85.7% to 68.5%, and overall antibiotic use with agents covering PSA also decreased significantly.^13^ While this study looked at patients systemwide, future studies looking at the impact of this order set specifically in KTRs is warranted, particularly given the high empiric anti-PSA antibiotic use seen in our study.

Our study identified two potential risk factors for PSA in KTRs: temporal proximity to transplantation and treatment for rejection within the year prior to DFI. As the greatest degree of immunosuppression in KTRs occurs in the immediate posttransplant period and during periods of treatment for rejection, we posit that the risk for PSA in DFI may be influenced by the degree of immunosuppression. This theory is supported by recent data in KTRs demonstrating high rates of infection following treatment for rejection, a rate which further increased in KTRs treated for multiple rejection episodes.^14^ A similar trend, though not statistically significant, was seen with thymoglobulin receipt in the year prior to DFI, in whom 40% of infections had PSA isolated. The lack of statistical significance could be due to insufficient sample size or may reflect that certain types of immunosuppression are more associated with risk for PSA than others. Further studies are needed to explore these particular possibilities.

Interestingly, a prior history of PSA infection was not associated with increased risk for subsequent DFI secondary to PSA (*P* = .311). Additionally, isolation of PSA was not associated with antibiotic receipt within the prior 30 days (*P* = .538). These results should be interpreted cautiously, however, due to our small sample size. Future, larger studies should aim to expound upon potential risk factors to discern a more targeted approach in antipseudomonal prescribing in this population.

PSA was not found to be a predictor of clinical severity in our study. However, nearly half (48.9%) of KTRs met criteria for severe DFI, highlighting the clinical significance of this infection in the KTR population. While transplant immunosuppression may contribute to this finding, our sample also consisted primarily of inpatients for whom culture-positive data were available, possibly conferring a bias towards severe cases.

Importantly, our study found a significant prevalence of DFI secondary to *Staphylococcus aureus* (30.4%), with close to half of isolates (45.8%) being methicillin-resistant. Similarly high rates of DFIs secondary to *Staphylococcus aureus* have been seen in the non-transplant patient population, including at our institution.^13,15^ These findings underscore the importance of ensuring empiric agents used for DFIs provide adequate anti-staphylococcal coverage, including coverage against MRSA (ie, vancomycin), with tailoring of therapy based on subsequent microorganism identification and susceptibilities. *Staphylococcus aureus* remains a highly virulent pathogen and should be treated expeditiously when clinically suspected.^16^

Our rate of DFI secondary to anaerobic organisms was also significant (16.5%). This is comparable to a reported anaerobic culture positive rate of 17% in a large review of DFI in non-transplant patients.^17^ The review further suggested the increased virulence of these pathogens, with *Bacteroides* species being associated with a higher relative rate of amputation.^17^ Therefore, consideration of empiric anaerobic coverage in certain KTR with DFI is reasonable pending microorganism identification.

There are several limitations to our study. As mentioned, our small PSA cohort may have limited our ability to identify risk factors for this microorganism. Additionally, we only included patients for whom microbiological data were available, which could have led to underreporting of certain microorganisms. We also included all tissue, fluid and wound cultures regardless of how they were obtained (surgically vs bedside), which may have led to misreporting colonizers or contaminants as true pathogens. Our study also only examined patients who were admitted for management of their DFIs, which is likely a more critically ill population compared to those managed in the outpatient setting. Finally, the microbiologic milieu of any institution is affected by local demographic and environmental factors, which may limit the generalizability of our results.

In conclusion, we found the prevalence of PSA to be low in DFIs in our KTRs but empiric prescribing of antipseudomonal agents to be high, suggesting an opportunity for antimicrobial stewardship in transplant infectious diseases. Temporal proximity to transplantation and treatment for rejection reflect possible risk factors for DFIs secondary to PSA and may be useful for risk stratification in guiding antipseudomonal antimicrobial therapy. Future, larger studies are needed to further identify risk factors and determine effective strategies to improve empiric prescribing.

## Research transparency and reproducibility

All data is available by request.
